# Adult intratesticular splenogonadal fusion simulating testicular cancer: a diagnostic blind spot in modern urologic oncology

**DOI:** 10.1093/jscr/rjag863

**Published:** 2026-09-29

**Authors:** Grace El Nabbout, John J Kowalczyk, Kelly J Manahan

**Affiliations:** Trinity School of Medicine, Kingstown, Saint Vincent and the Grenadines; Department of Urology, PIH Health, California, United States; Trinity School of Medicine, Kingstown, Saint Vincent and the Grenadines

**Keywords:** splenogonadal fusion, testicular mass, radical orchiectomy, testicular tumor mimic, preoperative diagnosis, urologic oncology

## Abstract

Splenogonadal fusion (SGF) remains one of the rarest and most diagnostically deceptive causes of scrotal pathology, frequently mimicking the clinical and imaging features of testicular malignancy despite its benign histology. Its exceptional rarity in adults, combined with the absence of reliable noninvasive preoperative diagnostic criteria, creates a major challenge in urologic practice, often necessitating management driven by concern for cancer. We present a 29-year-old man with a symptomatic left testicular mass and imaging highly suspicious for malignancy, prompting radical inguinal orchiectomy after standard evaluation could not confidently exclude a testicular neoplasm. Unexpectedly, final histopathology demonstrated ectopic splenic tissue consistent with SGF without evidence of germ cell tumour or other malignancy. This case highlights the persistent gap between preoperative suspicion and definitive diagnosis, emphasizing the importance of recognizing rare benign entities that closely imitate testicular cancer and may still lead to radical surgery in otherwise healthy young adults unnecessarily.

## Introduction

Splenogonadal fusion (SGF) is an exceptionally rare congenital anomaly caused by abnormal fusion of splenic tissue with the developing gonad or mesonephric derivatives during embryogenesis [1, 2]. Since its original classification by Putschar and Manion in 1956, <200 cases have been reported, underscoring its rarity and the limited clinical experience available to guide diagnosis and management [1, 2]. Because many patients remain asymptomatic or are diagnosed incidentally during surgery or histopathologic evaluation, the true prevalence is likely underestimated [2].

SGF is believed to arise between the fifth and eighth weeks of gestation, when the developing spleen lies adjacent to the left urogenital ridge. Abnormal attachment during this period may result in ectopic splenic tissue migrating with the descending gonad, explaining the marked left-sided predominance reported in the literature [1–3].

Two anatomic variants have been described. The continuous form maintains a fibrous or splenic connection to the native spleen and is frequently associated with congenital anomalies, including cryptorchidism, limb defects, and micrognathia. In contrast, the discontinuous form lacks continuity with the native spleen and typically presents as an isolated scrotal or intratesticular mass without associated malformations [1, 2, 4].

Although traditionally considered a pediatric condition, adult presentations remain diagnostically challenging because SGF frequently mimics testicular malignancy. Ultrasonography typically demonstrates a solid hypoechoic hypervascular intratesticular lesion resembling seminoma or other germ cell tumors [5–8].

We report a 29-year-old man with a palpable left testicular mass, suspicious imaging findings, and negative serum tumour markers who underwent radical inguinal orchiectomy. Final histopathology unexpectedly demonstrated SGF without malignancy, highlighting this persistent diagnostic blind spot in contemporary urologic oncology.

## Case

A 29-year-old man was referred for urologic evaluation after a left testicular mass was identified several months earlier during an emergency department visit. He reported intermittent dull discomfort of the left hemiscrotum without fever, weight loss, gross hematuria, or other systemic symptoms.

Physical examination revealed a palpable left testicular mass; the right testis was normal. Scrotal ultrasonography demonstrated a solid intratesticular lesion suspicious for malignancy. Serum tumor markers showed an alpha-fetoprotein (AFP) level of 2.1 ng/ml, negative beta-human chorionic gonadotropin (β-hCG), and lactate dehydrogenase (LDH) of 96 U/L.

Given the suspicious imaging findings, malignancy could not be excluded, and the patient underwent left radical inguinal orchiectomy. Through a standard inguinal approach, the spermatic cord was mobilized and ligated according to oncologic principles before delivery of the testis. A lesion involving the upper pole was identified with an intact tunica albuginea. Estimated blood loss was <1 ml, and no intraoperative complications occurred.

Gross examination demonstrated a 6.0 × 3.7 × 3.2 cm testis containing a well-circumscribed beefy-red hemorrhagic lesion measuring 2.7 × 2.0 × 1.5 cm. Histopathologic and immunohistochemical evaluation confirmed ectopic splenic tissue consistent with splenogonadal fusion. Immunostaining demonstrated CD20-positive B-cell follicles, CD3/CD8-positive T lymphocytes, CD163-positive macrophages, and CD21-highlighted follicular dendritic cell networks, supporting splenic differentiation. Additional findings included a benign Sertoli cell nodule, focal testicular microlithiasis, preserved spermatogenesis, and normal Leydig cells. No germ cell neoplasia in situ or invasive malignancy was identified. The postoperative course was uncomplicated.

## Discussion

SGF remains clinically significant not because of its frequency but because of its ability to closely mimic testicular malignancy. Although SGF is a benign congenital anomaly, adult presentations remain diagnostically challenging, often appearing clinically and radiographically indistinguishable from germ cell tumors [5, 6, 9]. Our patient presented with palpable unilateral intratesticular mass, suspicious ultrasonographic findings, and negative serum tumor markers, with the diagnosis established only after radical inguinal orchiectomy [5, 6, 8, 9].

Nearly seven decades after its original classification by Putschar and Manion, SGF continues to challenge the oncologic principle that any solid intratesticular mass should be presumed malignant until proven otherwise [1]. This approach remains appropriate because delayed treatment of testicular cancer may adversely affect outcomes. However, SGF represents a rare benign exception in which ectopic splenic tissue closely resembles malignancy. Kadouri et al. reported that most adult cases were diagnosed only after surgical excision, emphasizing that limited clinical recognition remains the greatest barrier to preoperative diagnosis [2].

Ultrasonography reliably detects intratesticular lesions but lacks sufficient specificity to distinguish SGF from malignancy. Typical findings include a solid hypoechoic hypervascular mass resembling seminoma. Seager et al. described branching internal vascularity resembling normal splenic architecture as a potential distinguishing feature, although this finding is subtle and inconsistently recognized [7]. Similar reports by Bat et al. and Zhou demonstrated lesions radiographically indistinguishable from testicular cancer until postoperative pathology confirmed SGF [8, 9]. Likewise, normal serum AFP and β-hCG levels provide limited reassurance because seminoma and early stage germ cell tumors frequently demonstrate similar laboratory findings [4–6]. Shen et al. also reported seminoma arising with SGF, illustrating that suspected SGF cannot reliably exclude concurrent malignancy [4].

Our patient’s management reflected current oncologic standards. Radical inguinal orchiectomy remains appropriate when malignancy cannot be confidently excluded using available clinical, laboratory, and imaging findings [5, 6]. Functional imaging with technetium-99 m sulfur colloid scintigraphy or single-photon emission computed tomography/computed tomography (SPECT/CT) and intraoperative frozen section analysis may facilitate diagnosis and testis sparing surgery in carefully selected patients, but only when SGF is considered preoperatively [10–12]. Greater awareness of this rare entity may improve diagnostic accuracy while preserving adherence to established oncologic principles.

## Conclusion

Splenogonadal fusion is a rare benign congenital anomaly that can closely mimic testicular malignancy. This case highlights the diagnostic challenges associated with adult intratesticular splenogonadal fusion. Greater awareness of this entity and consideration of additional diagnostic modalities in selected patients may facilitate preoperative recognition and potentially avoid unnecessary orchiectomy while maintaining oncologic safety.
